# The effects of native lactic acid bacteria on the microbiome, fermentation profile, and nutritive value of Napier grass silage prepared with different legume ratios

**DOI:** 10.3389/fmicb.2022.1112058

**Published:** 2023-01-30

**Authors:** Hao Guan, Haiping Li, Li Gan, Shiyong Chen, Yanhong Yan, Zhifeng Jia, Wenhui Liu, Xiaoxing Wei, Xiang Ma, Qingping Zhou

**Affiliations:** ^1^Sichuan Zoige Alpine Wetland Ecosystem National Observation and Research Station, Southwest Minzu University, Chengdu, China; ^2^Key Laboratory of Superior Forage Germplasm in the Qinghai-Tibetan Plateau, Qinghai Academy of Animal Science and Veterinary Medicine, Qinghai University, Xining, China; ^3^School of Mathematics and Statistics, Qinghai Normal University, Xining, China; ^4^College of Grassland Science and Technology, Sichuan Agricultural University, Chengdu, China

**Keywords:** lactic acid bacteria, Napier grass, alfalfa (*Medicago sativa* L.), mixed silage, microbial communities

## Abstract

Mixing grass with legumes before ensiling is beneficial for improving dry matter and crude protein yield, but additional information is needed to balance nutrient content and fermentation quality. In this study, the microbial community, fermentation characteristics, and nutrient content of Napier grass mixed with alfalfa at different proportions were assessed. Tested proportions included: 100:0 (M0), 70:30 (M3), 50:50 (M5), 30:70 (M7), and 0:100 (MF). Treatments included: (CK) sterilized deionized water; (IN) selected lactic acid bacteria: *Lactobacillus plantarum* CGMCC 23166 and *Lacticaseibacillus rhamnosus* CGMCC 18233 (1.5 × 10^5^ cfu/g of fresh weight for each inoculant); and (CO) commercial lactic acid bacteria: *L. plantarum* (1 × 10^5^ cfu/g of fresh weight). All mixtures were ensiled for 60 days. Data analysis was used as a completely randomized design with a 5-by-3 factorial arrangement of treatments. The results showed that with increasing alfalfa mixing ratio, dry matter, and crude protein increased, while neutral detergent fiber and acid detergent fiber decreased both before and after ensiling (*p* < 0.05), which was not influenced by fermentation. Inoculation with IN and CO decreased pH and increased the lactic acid content compared to CK (*p* < 0.05), especially in silages M7 and MF. The highest Shannon index (6.24) and Simpson index (0.93) were observed in the MF silage CK treatment (*p* < 0.05). The relative abundance of *Lactiplantibacillus* decreased with increasing alfalfa mixing ratio, while the abundance of *Lactiplantibacillus* was significantly higher in the IN-treated group than in other treatment groups (*p* < 0.05). A higher alfalfa mixing ratio improved the nutrient value, but also made fermentation more difficult. Inoculants improved the fermentation quality by increasing the abundance of *Lactiplantibacillus*. In conclusion, the groups M3 and M5 achieved the optimal balance of nutrients and fermentation. If a higher proportion of alfalfa needs to be used, it is recommended to use inoculants to ensure sufficient fermentation.

## Introduction

The increased demand for dairy products in many tropical and subtropical regions has highlighted the importance of the production and preservation of herbage (Namihira et al., 2010). As a perennial forage, Napier grass (*Pennisetum purpureum* Schum.) has been widely cultivated as ruminant feed in tropical and subtropical areas (Halim et al., 2013) because of its high yield as well as simple cultivation and management requirements. Alfalfa (*Medicago sativa* L.) is a high-quality forage legume with high protein content, which is generally not suitable for growing in humid and hot subtropical areas. However, with improved varieties and cultivation measures, it is being increasingly cultivated in subtropical areas.

When these herbages are produced for hay, during harvest season, the climate in subtropical areas is usually too rainy and humid for drying so that dry matter (DM) is often lost. Therefore, in such areas, ensiling is a more suitable and efficient strategy to conserve nutrients in herbage. However, Napier grass commonly has high moisture and fiber but low crude protein contents, which can easily lead to butyric acid fermentation and also offers little nutritional value for silage production (Halim et al., 2013; Guan et al., 2020). It is also difficult to produce high-quality silage using alfalfa because of its low water-soluble carbohydrate content and high buffer energy (Muck et al., 2007). Mixing grasses and legumes is a common type of mixed silage, as it can lessen disadvantages and utilize advantages of each component by complementing the characteristics of components themselves, thus improving the quality of silage overall.

In most studies on mixed silage, the grass component is either corn (Contreras-Govea et al., 2009, 2013; Zeng et al., 2020), sorghum (Lima et al., 2010; Lima-Orozco et al., 2014; Zhang et al., 2015), or oat (Chen et al., 2017), and the leguminous component is either alfalfa (Zhang et al., 2015; Chen et al., 2017) or soybean (Lima et al., 2010; Zeng et al., 2020). Mixing legume silage with grass silage should theoretically increase the crude protein (CP) concentration, but it would also increase other compounds, such as neutral detergent fiber (NDF), lactic acid, and the concentration of total acids, potentially decreasing digestibility (Contreras-Govea et al., 2011). Therefore, it is important to determine the proper legume and grass mixture ratio that will result in optimal nutritive value and fermentation. However, little research has been conducted on using Napier grass for silage mixed with a legume.

The objective of this study was to assess the effects of different legume and grass proportions and inoculants on nutritive value, fermentation characteristics, and microbial diversity of Napier grass and alfalfa mixed silage. The main hypothesis is that application of lactic acid bacteria (LAB) inoculants would result in higher silage quality than un-inoculated silage of Napier grass mixed with alfalfa at different ratios.

## Materials and methods

### Ensiling process

Napier grass [(*Pennisetum americanum × P. purpureum*) *× P. purpureum* Schum. cv. Guimu No. 1] and alfalfa (*M. sativa* L. cv. 6010) were grown on four plots within different blocks of an irrigated field at the National Grass Variety Regional Test Station, Xinjin, Chengdu, China (N30°76′, E103°76′). Napier grass was harvested when it reached the elongation stage at a height of 1.5–2 m, leaving a residual of 20–30 cm. Alfalfa were harvested by hand at full bloom in the third cutting, using a sickle and leaving a 5 cm stubble. After chopping to approximately 1–2 cm with a forage cutter (Lingong Machinery, Shandong, China), Napier grass and alfalfa were mixed at fresh weight ratios of 100:0, 70:30, 50:50, 30:70, and 0:100 (groups M0, M3, M5, M7, and MF, respectively). Groups of M0, M3, M5, M7, and MF were considered experimental units and treatments, which were applied in quadruplicate, were (1) sterilized deionized water (CK); (2) selected LAB: *Lactobacillus plantarum* CGMCC 23166 and *Lacticaseibacillus rhamnosus* CGMCC 18233 (1.5 × 10^5^ cfu/g of fresh weight of each inoculant) (IN); and (3) commercial LAB: *L. plantarum* (1 × 10^5^ cfu/g of fresh weight, purchased from Yaxin company) (CO). To ensure the viability of inoculants and appropriate inoculation rates, lactic acid bacteria (LAB) were plated as described below, counted before inoculation of forages, and then stored as recommended to retain viability and prevent multiplication. Forage from each experimental unit was ensiled (1 kg of forage) in two vacuum-heat sealed nylon-polyethylene standard barrier bags (0.09-mm thickness, 35 × 40 cm; Aodeju, China). Bags were stored in a temperature-controlled room (25–30°C) for 60 days, so that four replicate bags per treatment per ensiling duration were obtained. Air was withdrawn from silos immediately before they were sealed with an external clamp vacuum pump (OX600P, OUXIN Vacuum Pump Equipment Manufacturing Co., Ltd., China).

### Chemical, fermentation, and microbial analyses

Extracts of silage samples from d 60 were obtained by mixing 20 g of silage with 180 ml of 0.1% peptone water in a stomacher (Lab-blender 400; Tekmor company) for 1 min. The solution was filtered through two layers of sterilized cheesecloth, and one aliquot was immediately used for measuring total LAB, yeast, mold, and *Enterobacter* counts. Briefly, the amount of LAB was quantified by MRS Agar (CM 188, Land Bridge, Beijing, China). Molds and yeasts were cultured on potato dextrose agar (CM 123, Land Bridge, Beijing, China), and *Enterobacter* bacteria were quantified using violet red bole agar (CM 115, Land Bridge, Beijing, China). Another silage extracted aliquot was used for measuring pH (PHSJ-5; LEICI, Shanghai, China). After pH measurement, 40 ml of silage extracted aliquots was acidified with 1% of 50% sulfuric acid, and centrifuged at 7,000 ×*g* for 15 min at 4°C. One part of the supernatant was retained for ammonia-N analysis (Kozloski et al., 2006) and another part was filtered through 0.22-μm filters and transferred to vials for organic acids analysis. Organic acids (i.e., lactic acid, acetic acid, propionic acid, and butyric acid) of 45 silage samples were then analyzed by high-performance liquid chromatography with a UV detector (210 nm) and a column (KC-811, Shimadzu Co., Ltd., Kyoto, Japan) according to Guan et al. (2018).

Samples from 0 and 60 d were dried at 60°C for 48 h in a forced-air oven, ground by a grinder (CT293 Cyclotec™, FOSS Analytical A/S, Hillerød, Denmark), and passed through a 1-mm mesh sieve. Then, samples were analyzed for DM, ash (method 942.05; AOAC International, 2012), NDF, and Acid detergent fiber (ADF) without correction for residual ash [Ankom 200 Fiber Analyzer, Ankom Technology; methods 2002.04, AOAC International (2012) for NDF and 973.18, AOAC International (2006) for ADF], CP (Kjeldahl method, method 990.03, AOAC International, 2006), and water-soluble carbohydrates (WSC). The content of WSC in the sample was detected by Micro Plant Soluble Sugar Content Assay Kit (BC0035, Beijing Solarbio Science & Technology Co., Ltd., Beijing, China).

### Microbiome analysis

The method of DNA extraction was described in (Guan et al., 2018). A total of 50 g frozen sample was passed through a 4 mm sieve after freeze-drying and smashing. A subsample (5 g) was ball milled for 1 min at room temperature and total DNA was extracted *via* the TIANamp Bacteria DNA isolation kit (DP302-02, Tiangen, Beijing, China). All samples were purified *via* purification and recovery of the DNA kit column (DP214-02, Tiangen, Beijing, China) and purified samples were then eluted in nuclease free water. NanoDrop 2000 was used to measure the purity and concentration of DNA. Qualifying DNA samples were stored at-20°C for future analysis.

16S rRNA genes of distinct regions (16S V4) were amplified using the specific primers 515 F (5’-GTTTCGGTGCCAGCMGCCGCGGTAA-3′) and 806R (5’-GCCAA TGGACTACHVGGGTWTCTAAT-3′). Samples including a bright main strip between 400 and 450 bp were chosen for further experiments. Sequencing libraries were generated and sequenced as described by Guan et al. (2018). Illumina S5 sequencing was performed by Novogene (Beijing, China) according to the manufacturer’s recommendations.

### Sequence analyses

NGS reads were assembled using FLASH (version 1.2.7; Magoč and Salzberg, 2011). Low-quality reads were excluded according to the QIIME quality control process (version 1.7.0; Caporaso et al., 2010). Chimeric sequences were removed by using the UCHIME algorithm to obtain final effective tags (Edgar, 2013). Uparse software (version 7.0.1001) was used for sequence analyses (Edgar, 2013). Operational taxonomic units were defined by a 97% similarity cutoff. To annotate taxonomic information, the Silva Database^1^ (Quast et al., 2012) was used based on the Mothur algorithm. Alpha diversity metrics (Observed-species, Chao1, Shannon, Simpson, ACE, and Good-coverage) and beta diversity metrics (weighted UniFrac and unweighted UniFrac) were calculated with QIIME software (version 1.7.0). Principal coordinate analysis was conducted with R software (version 2.15.3). The sequence data reported in this study have been submitted to the NCBI database (PRJNA884491).

### Statistical analyses

Microbe populations were estimated as cfu/g and values were log transformed prior to statistical evaluation. All analyses were conducted using the general linear model procedure in SPSS 22. Data related to different proportions of alfalfa and Napier grass prior to ensiling, fermentation indexes, chemical composition, microbial population, and alpha diversity indexes of silage were subjected to two-way ANOVA with the following model:


$$Y_{ij}=\mu +\alpha_{i}+\beta_{j}+{\left(\alpha \times \beta \right)}_{ij}+e_{ij}\text{,}$$

where *Y_ij_* is the dependent variable, *μ* is the overall mean, *α_i_* is the fixed effect of proportion, *β_j_* is the effect of inoculants, *(α × β)_ij_* is the interaction between proportion and inoculants, and *e_ij_* is the residual error. Differences among treatment means were separated by using Tukey’s honest significant difference test, and significance was declared at *p* < 0.05.

## Results

### Chemical compositions of Napier grass, alfalfa, and their mixture before ensiling

The nutrient compositions of mixes of alfalfa and Napier grass at different proportions prior to ensiling are shown in Table 1. The mixing proportion had extremely significant impacts on DM, CP, ash, NDF, and ADF (*p* < 0.01). The DM content of Napier grass was only 19.33%, while that of alfalfa was 32.53%. With increasing alfalfa proportion in the mixture, the DM content increased significantly (*p* < 0.01). The lowest CP was found in M0, and CP increased significantly with increasing alfalfa proportion (*p* < 0.01). The contents of WSC, ash, NDF and ADF followed a significant downward trend with increasing alfalfa proportion (*p* < 0.01).

**Table 1:** Nutrient composition of different proportion of Napier grass and alfalfa prior to ensiling.

Proportion	DM (%)	CP (% DM)	WSC (% DM)	Ash (% DM)	NDF (% DM)	ADF (% DM)
M0	19.33d	8.67d	6.96a	11.86a	65.78a	36.76a
M3	22.54c	13.67c	5.99ab	10.85b	58.49b	35.93a
M5	28.39b	14.89b	5.42ab	9.79c	53.33b	34.51a
M7	29.40b	15.97a	4.55b	9.51c	52.90b	30.46a
MF	32.53a	16.34ab	3.55c	8.42d	48.95c	27.08b
SEM	0.21	0.38	1.46	0.25	2.91	2.63
Significance	**	**	0.032*	**	0.008^**^	**

^a–d^Means in the same column followed by different alphabets are significantly different (*p < 0.05, **p < 0.01). DM, dry matter; SEM, standard error of mean.

### Fermentation parameters and microbial counts of mixed alfalfa and Napier grass silage

As shown in Table 2, proportions, inoculants, and their interactions had extremely significant effects on pH and NH_3_-N [% total nitrogen (TN)]. The pH and NH_3_-N (%TN) increased with increasing proportion of alfalfa. The pH of the CK silage was significantly higher than pH levels of IN- and CO-treated silage except for Group M0 (*p* < 0.05). In Group M0, there was no significant difference among treatments (*p* > 0.05). The highest pH of 4.69 was observed in MF without treatment (CK). A similar tendency was found in NH_3_-N (%TN), where MF without treatment (CK) had the highest NH_3_-N (%TN), which exceeded 10% (12.37%). M0 silage treated with IN and CO significantly decrease NH_3_-N (%TN) compared to CK. Proportions (*p* = 0.017) and inoculants (*p* = 0.023) had significant effects on the concentration of lactic acid (LA), while the interaction had an extremely significant effect on LA concentration (*p* = 0.002). IN-treated silages had higher concentrations of LA than CK-and CO-treated silages in every proportion expect Group M0. The lowest concentration of LA was observed in MF silage without treatment (CK). Proportion and inoculants had an extremely significant effect on concentration of acetic acid (AA, *p* < 0.01), while no significant effect was found in their interaction (*p* > 0.05). The acetic acid content of all treatments did not exceed 20 g/mg DM. Propionic acid was not detected in any of the silages. A small amount of butyric acid was detected in individual silages, but this was observed in all silages of the MF group (Table 2).

**Table 2:** Effects of mixed proportion and inoculants on fermentation indexes of alfalfa and Napier grass mixed silage.

Treatment		pH	NH_3_-N (%TN)	Lactic acid (g/mg DM)	Acetic acid (g/mg DM)	Lactic acid/acetic acid	Propionic acid (g/mg DM)	Butyric acid (g/mg DM)
Proportion	Inoculants							
M0	CK	4.00fg	4.51gh	109.48ab	5.61e	19.51a	ND	ND
	IN	3.95g	4.39gh	85.47abc	7.30de	11.70b	ND	ND
	CO	3.96g	4.60fgh	99.50ab	6.40de	15.54ab	ND	ND
M3	CK	4.09cdef	5.10efg	96.10ab	9.20bcde	10.44b	ND	0.32
	IN	3.94g	3.23i	120.59a	8.20cde	14.70ab	ND	ND
	CO	3.96 g	3.73 h	97.50ab	9.60bcde	10.15b	ND	0.63
M5	CK	4.13bcd	5.22efg	97.82ab	10.14bcde	9.64b	ND	0.64
	IN	4.02efg	5.76cde	106.66ab	12.10abce	8.81bc	ND	ND
	CO	4.04defg	5.53def	71.42bc	10.11abcde	7.06bc	ND	ND
M7	CK	4.24b	6.43bcd	79.66bc	12.42abcde	6.41c	ND	0.63
	IN	4.10cdef	7.09b	128.50a	13.46abcd	9.54b	ND	ND
	CO	4.12cde	6.61bc	104.34ab	15.80abc	6.60c	ND	ND
MF	CK	4.69a	12.37a	35.87d	16.42ab	2.18f	ND	1.08
	IN	4.16bc	5.09efg	89.67abc	17.92a	5.0d	ND	0.84
	CO	4.24b	7.20b	53.65bc	11.8abcde	4.5de	ND	0.77
SEM		0.05	0.305	0.40	0.32	0.23	-	-
Proportion		**	**	0.017*	**	*	-	-
Inoculants		**	**	0.023*	**	*	-	-
Proportion × Inoculants		**	**	0.002**	NS	**	-	-

^a–i^Means in the same column followed by different alphabets are significantly different (*p < 0.05, **p < 0.01). NS, not significant; SEM, standard error of mean; DM, dry matter; TN, total nitrogen; ND, not detected.

As shown in Table 3, yeast, mold, and *Enterobacter* could not be detected in the silage after 60 days of anaerobic fermentation, except for CK. Totals of 2.95 Log cfu/g FM of mold and 2.32 Log cfu/g FM of *Enterobacter* were detected in CK. Proportion, inoculants, and their interaction had extremely significant effects on LAB counts. Silage inoculated with IN had a significantly higher number of LAB than CK and CO in Groups M0, M3, M7, and MF (*p* < 0.05).

**Table 3:** Effects of mixed proportion and inoculants on chemical composition of alfalfa and Napier grass mixed silage.

Treatment		LAB (log cfu/g FM)	Molds (log cfu/g FM)	Yeast (log cfu/g FM)	Enterobacter (log cfu/g FM)
Proportion	Inoculants				
M0	CK	2.43e	ND	ND	ND
	IN	3.70c	ND	ND	ND
	CO	1.44g	ND	ND	ND
M3	CK	2.31e	ND	ND	ND
	IN	4.25b	ND	ND	ND
	CO	1.89f	ND	ND	ND
M5	CK	5.41a	ND	ND	ND
	IN	3.50c	ND	ND	ND
	CO	1.59fg	ND	ND	ND
M7	CK	2.64e	ND	ND	ND
	IN	3.53c	ND	ND	ND
	CO	1.35g	ND	ND	ND
MF	CK	0.26h	2.95	ND	2.32
	IN	3.10d	ND	ND	ND
	CO	1.69fg	ND	ND	ND
SEM		0.115	-	-	-
Proportion		**	-	-	-
Inoculants		**	-	-	-
Proportion × Inoculants		**	-	-	-

^a–g^Means in the same column followed by different alphabets are significantly different (*p < 0.05, **p < 0.01). SEM, standard error of mean; FM, fresh matter; ND, not detected.

### Chemical composition of mixed alfalfa and Napier grass silage

Table 4 shows the effects of mixing proportion and inoculants on chemical composition of mixed alfalfa and Napier grass silage. Proportion had extremely significant effects on DM, DM loss, CP, ash, NDF, and ADF (*p* < 0.01). Interaction with inoculants had extremely significant effects on DM and ash (*p* < 0.01), while inoculants had no significant effect on DM, CP, ash, NDF, and ADF (*p* > 0.05). DM, CP, ash, NDF, and ADF in silages showed similar trends with forage at different proportions. DM and CP increased with increasing alfalfa proportion, while NDF and ADF decreased. WSC decreased after ensiling, and inoculants had an extremely significant effect on WSC (*p* < 0.01), but proportion and their interaction had no significant effects (*p* > 0.05).

**Table 4:** Effects of mixed proportion and inoculants on microbial quantity of alfalfa and Napier grass mixed silage.

Treatment		DM (%)	DM loss (%)	CP (% DM)	Ash (% DM)	NDF (% DM)	ADF (% DM)	WSC (% DM)
Proportion	Inoculants							
M0	CK	20.91k	0.31bc	8.92f	9.74b	54.25a	29.72a	1.29bc
	IN	22.30j	0.34bc	8.08f	9.95a	53.07a	29.02a	1.94abc
	CO	22.2ij	0.17c	8.05f	9.69b	54.52a	28.53ab	1.43a
M3	CK	24.19i	0.39bc	13.39de	8.79cd	54.87a	29.94a	1.01bc
	IN	25.38h	0.31bc	14.34abcde	8.72cde	52.92ab	28.83a	0.75c
	CO	25.36h	0.25bc	14.14bcde	8.90c	54.14a	27.13a	3.61ab
M5	CK	28.34f	0.50bc	13.74cde	8.43fg	50.33b	26.79ab	1.31bc
	IN	29.37e	0.22bc	14.27abcde	8.32gh	52.21b	26.77ab	2.90abc
	CO	27.57g	0.31bc	13.94cde	8.65de	50.91b	25.91ab	3.92a
M7	CK	30.62d	0.46bc	15.18abcde	8.68de	50.55c	26.07ab	1.90abc
	IN	32.53c	0.37bc	16.56a	8.33gh	52.66ab	27.17ab	1.87abc
	CO	30.67d	0.32bc	15.52abcd	8.17hi	52.58ab	27.68ab	1.03bc
MF	CK	32.57c	1.03a	16.65abcde	8.56ef	48.80c	25.36b	1.04bc
	IN	34.53a	0.39bc	16.07abc	8.63de	49.74c	25.35b	2.07abc
	CO	33.40b	0.54b	16.37ab	8.03i	49.84c	25.96b	2.12abc
SEM		0.193	0.14	1.02	0.09	2.52	2.72	0.19
Proportion		**	**	**	**	**	**	NS
Inoculants		NS	**	NS	NS	NS	NS	**
Proportion × Inoculants		**	NS	NS	**	NS	NS	NS

^a–j^Means in the same column followed by different alphabets are significantly different (*p < 0.05, **p < 0.01). NS, not significant; SEM, standard error of mean; DM, dry matter; TN, total nitrogen; ND, not detected.

### Microbial silage diversity

As shown in Table 5, Shannon and Simpson indexes were extremely significantly different between proportion and inoculants (*p* < 0.01), and the Simpson index of their interaction was extremely significantly different (*p* < 0.01). The highest Shannon index (6.24) was observed in MF silage without inoculants (*p* < 0.05). Inoculants and their interaction with proportion showed no significant difference in Chao1 (*p* > 0.05). Chao1 and Ace indexes were significantly different for proportion (*p* < 0.05).

**Table 5:** Effects of mixed proportion and inoculants on microbial alpha diversity index of alfalfa and Napier grass mixed silage.

Treatment		Shannon	Simpson	Chao 1	Ace
Proportion	Inoculants				
M0	CK	4.13cdef	0.80c	1206.73bcd	1289.79bcde
	IN	3.35f	0.69d	773.26cd	802.78de
	CO	3.43ef	0.64d	843.75cd	860.96cde
M3	CK	4.81bc	0.82bc	1307.98abcd	1361.51bcde
	IN	3.61def	0.70d	981.78bcd	1095.18bcde
	CO	4.63bcd	0.81c	867.37 cd	931.06cde
M5	CK	4.51bcde	0.84bc	888.64 cd	950.35cde
	IN	3.44ef	0.68d	995.42bcd	1043.55bcde
	CO	4.89bc	0.90ab	1025.31bcd	1114.67bcde
M7	CK	4.58bcd	0.82c	1473.34abc	1587.18abc
	IN	4.76bc	0.83bc	1394.84abcd	1518.10abcd
	CO	5.40b	0.86abc	1598.84ab	1695.39ab
MF	CK	6.24a	0.93a	1917.33a	2055.53a
	IN	4.549bcde	0.85bc	746.90d	778.36e
	CO	4.50bcde	0.81c	859.28cd	874.99cde
SEM		0.47	0.04	292.98	305.49
Proportion		**	**	*	*
Inoculants		**	**	NS	NS
Proportion × Inoculants		*	**	NS	*

^a–f^Means in the same column followed by different alphabets are significantly different (*p < 0.05, **p < 0.01). NS, not significant; SEM, standard error of mean.

**Figure 1:**
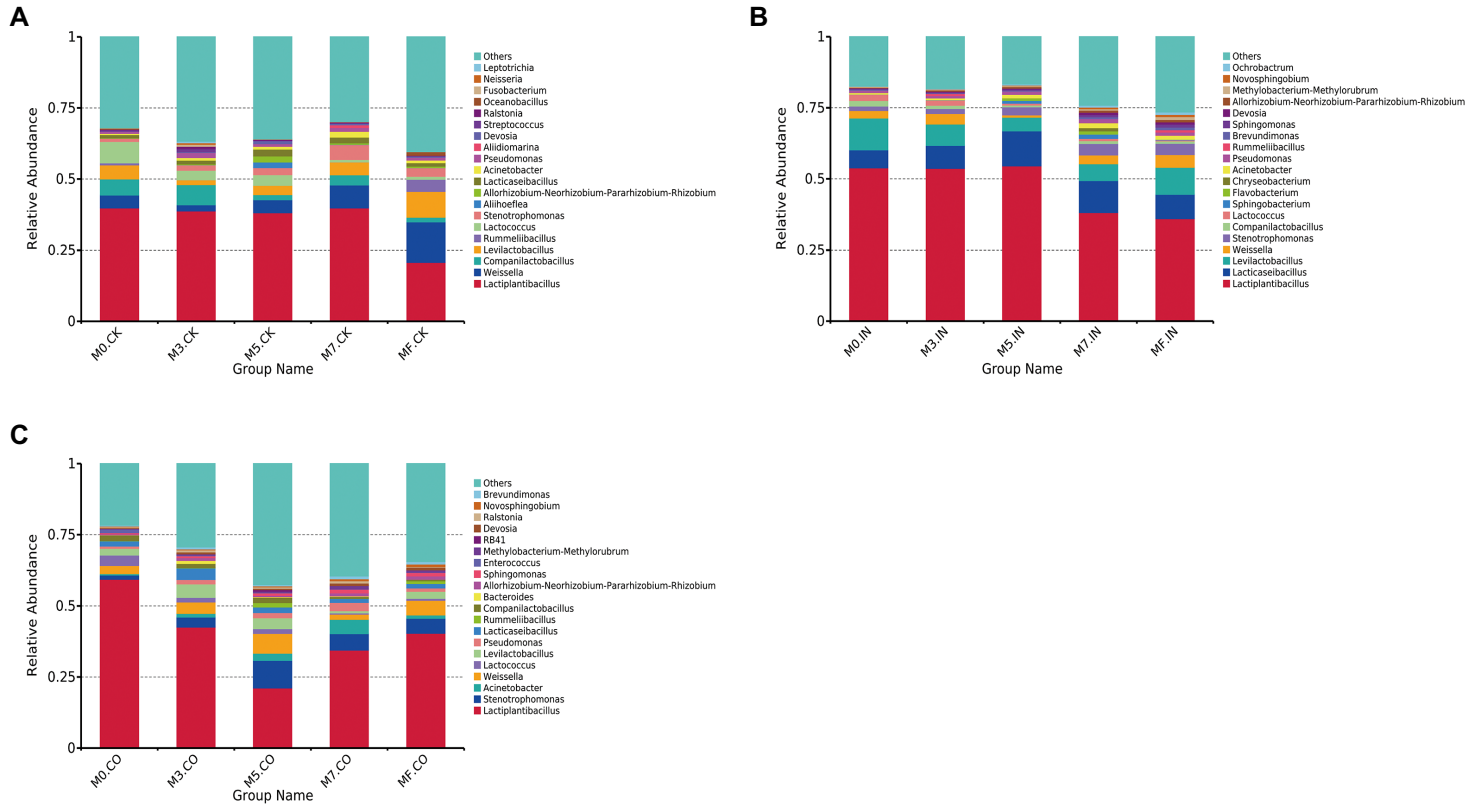
The bacterial relative abundances of mixed Napier grass and alfalfa silage at different mixing proportions under the same treatment (at genus level). **(A)** Bacterial relative abundances of mixed silage at different proportions without inoculation. **(B)** Bacterial relative abundances of mixed silage at different proportions with IN. **(C)** Bacterial relative abundances of mixed silage at different proportions with CO.

The microbial relative abundances of mixed Napier grass and alfalfa silage at different mixing proportions under the same treatment are shown in Figure 1. *Lactiplantibacillus*, *Weissella*, and *Companilactobacillus* were the most dominate bacterial genera across different proportions of mixed silage without inoculants (Figure 1A). The relative abundance of *Lactiplantibacillus* in all silages did not exceed 50%. The lowest *Lactiplantibacillus* and the highest *Weissella* abundances were observed in MF silages without inoculants (*p* < 0.05). The three genera with the highest relative abundances were *Lactiplantibacillus*, *Lacticaseibacillus*, and *Levilactobacillus* in silages treated with IN (Figure 1B). The relative abundance of *Lactiplantibacillus* exceeded 50% in M0, M3, and M5 silages treated with IO. *Lactiplantibacillus*, *Stenotrophomonas*, and *Acinetobacter* were dominant bacteria in silages treated with CO (Figure 1C). The relative abundance of *Lactiplantibacillus* did not exceed 50% except for M0 silage treated with CO. M5 silages treated with CO had lowest relative abundance of *Lactiplantibacillus*, which did not exceed 25%.

**Figure 2:**
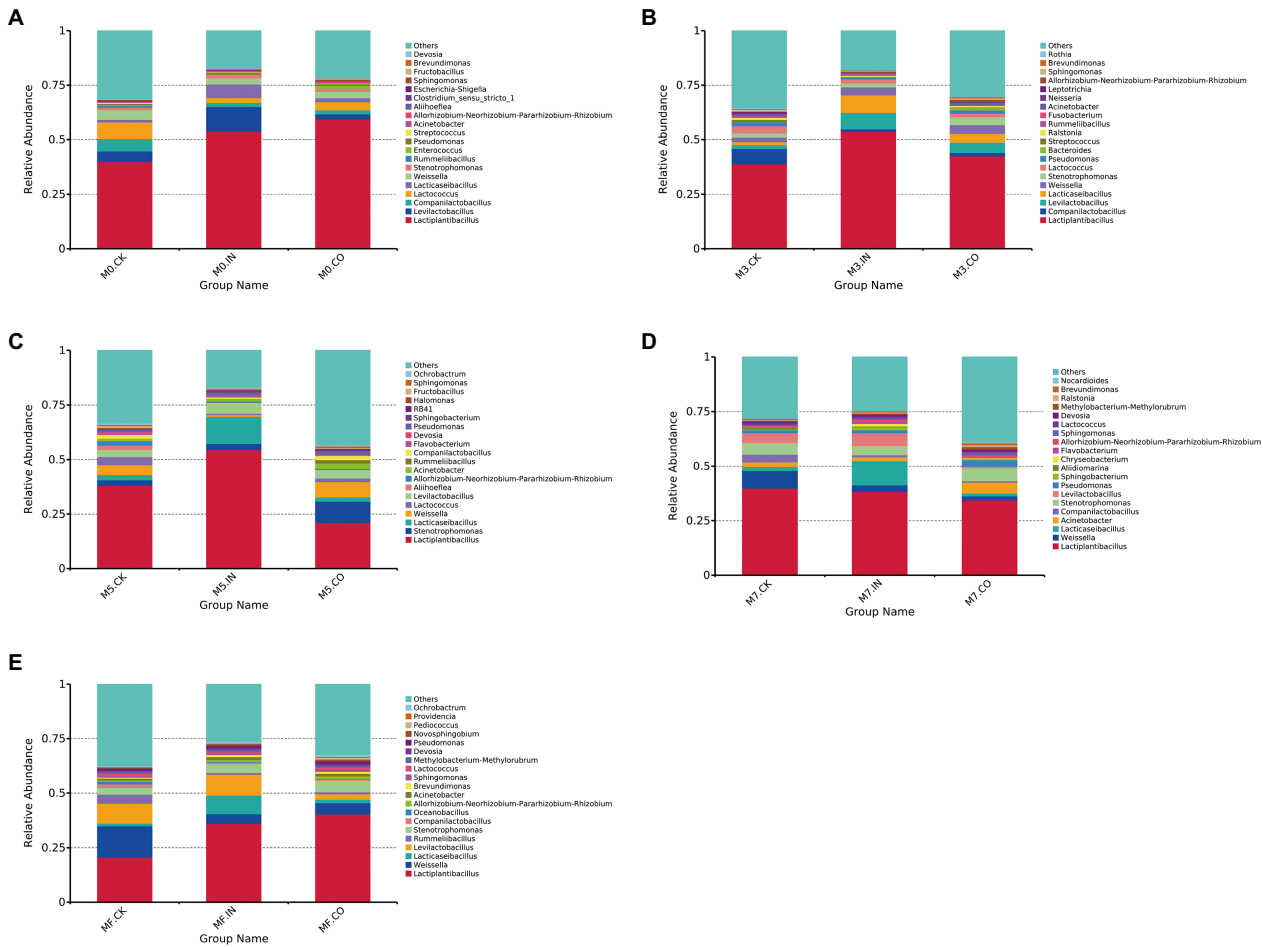
The relative microbial abundance of mixed Napier grass and alfalfa silage with different treatments but under the same proportion (at genus level). **(A)** Bacterial relative abundances of mixed silage with different treatments at M0. **(B)** Bacterial relative abundances of mixed silage with different treatments at M3. **(C)** Bacterial relative abundances of mixed silage with different treatments at M5. **(D)** Bacterial relative abundances of mixed silage with different treatments at M7. **(E)** Bacterial relative abundances of mixed silage with different treatments at MF.

The relative microbial abundance of mixed Napier grass and alfalfa silage with different treatments but under the same proportion is shown in Figure 2. The relative abundance of *Lactiplantibacillus* increased in M0 silages both with IN and M0 inoculation, both exceeding 50% (Figure 2A). The highest abundance of *Levilactobacillus* was found in M0 treated with IN. The relative abundance of *Lactiplantibacillus* showed a similar trend in M3 silages treated with IN and M0 (Figure 2B). The highest relative abundance of *Lactiplantibacillus* was found in M3 and M5 silages with IN (Figures 2B,C), which exceeded 50%. The relative abundance of *Lactiplantibacillus* decreased increasing proportion of alfalfa, which did not exceed 50% in all M7 and MF silages. The highest relative abundance of *Lactiplantibacillus* was found in M7 and MF silages treated with IN, while M7 and MF silages without inoculants had the highest relative abundance of *Weissella*.

**Figure 3:**
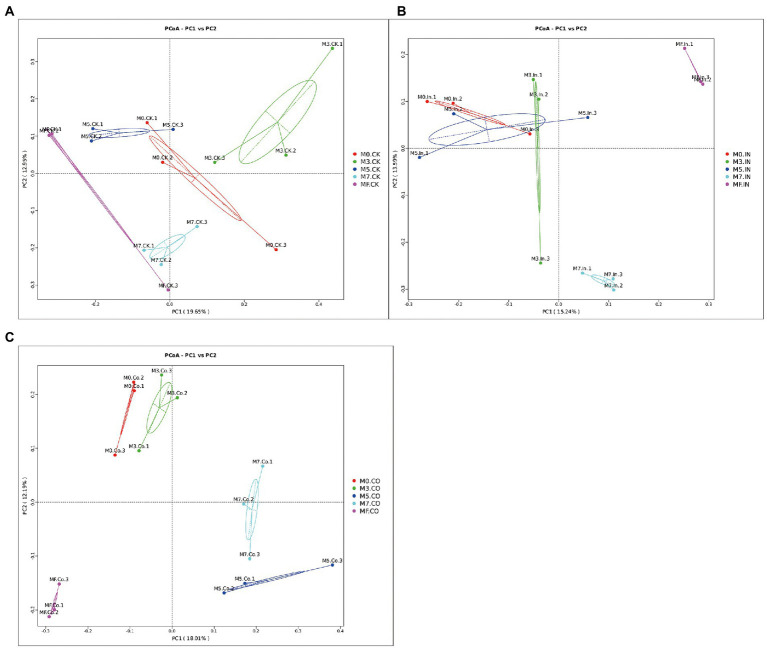
Principal coordinate analysis (PCoA)of mixed Napier grass and alfalfa silage with different mixing proportions under the same treatment. **(A)** PCoA of mixed silage at different proportions without inoculation. **(B)** PCoA of mixed silage at different proportions with IN. **(C)** PCoA of mixed silage at different proportions with CO.

The results of principal coordinate analysis of mixed Napier grass and alfalfa silage with different mixing proportions under the same treatment are shown in Figure 3. Bacterial communities were clearly separated between different proportions of silages with or without CO (Figures 3A,C). As shown in Figure 3B, although M0, M3, and M5 silages treated with IN can be pooled, these three groups cannot be easily separated from each other. However, they can easily be separated from M7 and MF silages treated with IN, and maintain a certain distance.

**Figure 4:**
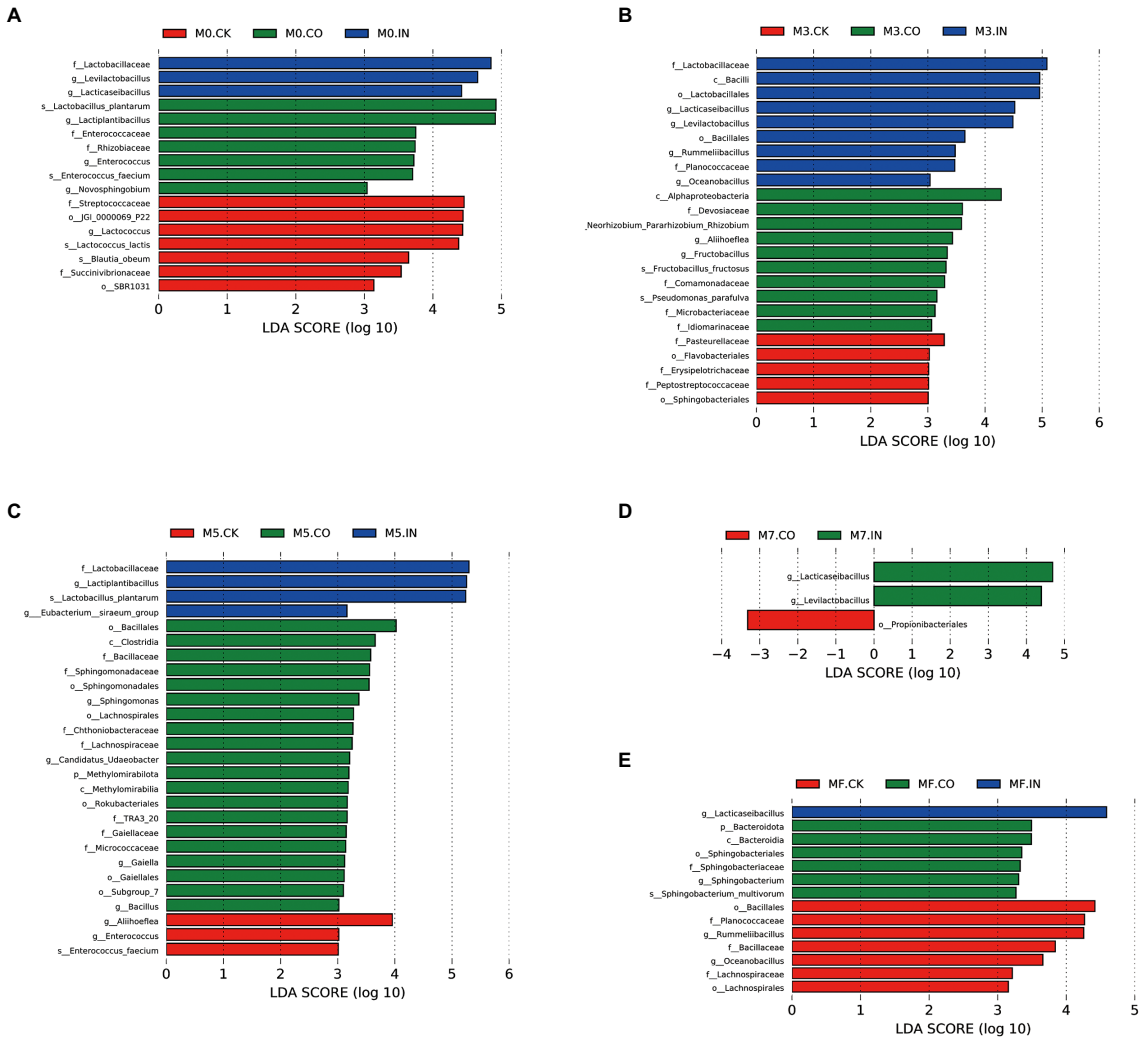
The differences in bacterial communities between different treatments under the same mixing proportion using LDA effect size (LEfSe) analysis (LDA > 3, *p* < 0.05). **(A)** LEfSe analysis of mixed silage with different treatments at M0. **(B)** LEfSe analysis of mixed silage with different treatments at M3. **(C)** LEfSe analysis of mixed silage with different treatments at M5. **(D)** LEfSe analysis of mixed silage with different treatments at M7. **(E)** LEfSe analysis of mixed silage with different treatments at MF.

Figure 4 shows the differences in bacterial communities between different treatments under the same mixing proportion using LDA effect size (LEfSe) analysis (LDA > 3, *p* < 0.05). The genera *Levilactobacillus* and *Lactiplantibacillus* were significantly different (biomarker) in M0 silages treated with IN, while *Lactiplantibacillus* was the biomarker in M0 silages treated with CO (Figure 4A). *Levilactobacillus* and *Lactiplantibacillus* were still biomarkers in M3 silages treated with IN (Figure 1B), while *Alphaproteobacteria* and *Pasteurellaceae* were biomarkers in M3 silages with and without CO inoculation, respectively. *Lactiplantibacillus* was still the biomarker in M5 silages treated with IN (Figure 4C), while Bacillales and Aliihoeflea were biomarkers in M5 silages with or without CO inoculation, respectively. *Lacticaseibacillus* and *Levilactobacillus* were biomarkers in M7 silages treated with IN (Figure 4D), while Propionibacteriales was the biomarker in M7 silages treated with CO. *Lacticaseibacillus* was still the biomarker in MF silages treated with IN (Figure 4E), while Bacteroidota and Bacillales were biomarkers in MF silages with or without CO inoculation, respectively.

## Discussion

### Nutritive value before ensiling

The DM content at the time of ensiling was at the appropriate level for alfalfa (M0) but was below 20% for Napier grass. This result is consistent with the research of Guan et al. (2020) and Widiyastuti et al. (2014), where the moisture content of Napier grass often exceeded 80% at harvest. With increasing proportion of alfalfa, the DM content of mixed forage increased significantly. However, although the proportion of alfalfa and Napier reached 70:30 (MF), their DM content was lower than that recommended (30%) for ensiling (Mcdonald et al., 1991). The CP content increased with increasing proportion of alfalfa in the mixture. This response agrees with previous research (Dawo et al., 2007; Contreras-Govea et al., 2009, 2011). Napier grass is by nature low in CP. Therefore, it can be expected that adding a legume such as alfalfa (which is high in CP) would increase CP in the mixture. NDF, ADF, and WSC followed a downward trend with increasing alfalfa mix proportion, which is due to the lower NDF, ADF, and WSC contents of alfalfa compared with Napier grass.

### Nutritive value after ensiling

While the CP content among mixtures was similar between pre-ensiled and ensiled mixtures, this was not the case for the NDF content. The NDF content was higher in pre-ensiled than in ensiled mixtures, which agrees with the results of Contreras-Govea et al. (2011) and Chen et al. (2017). However, Contreras-Govea et al. (2009) reported greater NDF contents in fermented than in pre-ensiled treatments when mixing corn with various climbing beans. Generally, it is assumed that cell wall structural carbohydrates are not affected during fermentation and LAB are consuming the pool of WCS. WSC depletion during fermentation would increase the NDF content after fermentation. To assess the effect fermentation had on cell wall structure, Jones et al. (1992) ensiled alfalfa under two DM contents (29 and 40.1%), with and without inoculants. They reported that low DM content and low pH predispose to hydrolysis of cell wall structural carbohydrates. They also found that arabinose and galactose decreased under both DM contents, while rhamnose only decreased under the lower DM content. All these carbohydrates were part of the hemicellulose fraction, which is included in the NDF content. Even though Jones et al. (1992) did not find hydrolysis of cellulose, they reported that a previous study observed hydrolysis of the cellulosic fraction in alfalfa (Morrison, 1989), which is part of the ADF fraction. Rooke and Hatfield (2003) reported that ensiling conditions impact carbohydrate pools. They also suggested that most of the changes that occurred in the pectic and hemicellulosic fraction may be related to the arabinosylic fraction, which is linked to xylose and galactose. These structural carbohydrates are susceptible to acid hydrolysis even under weakly acidic conditions (Rooke and Hatfield, 2003). In the present study, silage conditions were adequate for cell wall hydrolysis as reported by Jones et al. (1992). Therefore, it is likely that acidic conditions were conducive to cell wall hydrolysis, which decreased NDF content and, to some degree, also ADF content after ensiling. The WSC of all silage decreased after fermentation because of consumption by microorganisms, and it was therefore no longer affected by the mixing ratio.

### Fermentation characteristics

It has been well documented that legume silages such as alfalfa, fava bean, soybean, pea, red clover (*Trifolium pratense* L.), and kura clover have pH levels that range from 4.0 to 4.8 and are high in NH_3_-N (Owens et al., 1999; Mustafa and Seguin, 2003) as a result of high buffering capacity and extended fermentation. In the current study, pH increased with increasing alfalfa proportion in mixed silage. These results agree with previously reported findings (Contreras-Govea et al., 2009; Lima et al., 2010). Inoculation with LAB can increase the lactic acid content and reduce the pH (Muck et al., 2018), which is consistent with the results of M5, M7, and MF silages inoculated with IN and CO. Warm-season grasses often have a higher moisture content at harvest than cereal crops, which more easily results in lower pH levels (Bernardes et al., 2018). This is why the pH levels of M0 and M3 silages were not significantly different with or without inoculants in the current study. It has been suggested that a 2.5–3:1 lactic acid:acetic acid ratio is a good indicator of homolactic fermentation (Mcdonald et al., 1991). It is commonly assumed that a lower lactic acid:acetic acid ratio indicates that the fermentation is more heterolactic. In the present study, the lactic acid-to-acetic acid ratio decreased with increasing alfalfa content in the mixture. This could have both positive and negative impacts. The positive impact is that adding alfalfa increases the acetic acid concentration, which could result in better aerobic stability of Napier grass-alfalfa mixtures. The negative impact is that epiphytic LAB of alfalfa are normally heterofermentative LAB, which produce high acetic acid concentrations (Lin et al., 1992). Adding alfalfa to Napier grass therefore potentially increases the acetic acid concentration to a level (>40 g kg^−1^ DM) that could reduce the degree of pH decline, and affect both palatability of the silage and DM intake (Kung et al., 2018). Silages (especially legume silages) treated with a homolactic acid inoculant have a slightly higher lactic acid to acetic acid ratio (Kung et al., 2018) because homofermentative LAB produce lactic acid only. This is inconsistent with the data of the present study for MF silage treated with IN and CO. NH_3_-N increased with increasing proportion of alfalfa in mixed silages. These results were expected because legumes commonly have a high CP concentration, which is more conducive to proteolysis than the CP concentration of Napier grass. In addition, legumes have a better buffering capacity than grasses (Buxton and O'Kiely, 2003). As a consequence, higher NH_3_-N formation should be expected in the mixture as the proportion of legumes increases (Mcdonald et al., 1991). NH_3_-N of total N in most silages is below 10%, but alfalfa silage with high moisture content generally has 10–15% NH_3_-N, which is consistent with MF silage without treatment (Kung et al., 2018).

### Microbial populations

Generally, following anaerobic fermentation, the complex microbial communities of raw materials are gradually replaced by LAB, which is one of the criteria for successful silage (Mcdonald et al., 1991). Therefore, the microbial diversity will decrease sharply after successful fermentation. In the current study, MF silage without inoculant had the highest Shannon index, which is consistent with the data for the poorest fermentation quality of MF silage without inoculant. Different from the fermentation process of corn or grass silage, *Lactobacillus* can quickly occupy the absolutely dominant position, thus reducing silage microbial diversity (Guan et al., 2018). Legume forages are not easy to ensile, as undesirable microorganisms like *Clostridia*, *Bacillus*, and *Enterobacter* always cause butyric acid accumulation and proteolysis during ensiling (Silva et al., 2016). This can explain why the Shannon index of alfalfa and the proportion of mixed alfalfa and Napier grass silage were higher in other treatment groups. *Clostridium* fermentation is often found in alfalfa silage, resulting in higher butyric acid contents (Zheng et al., 2017; Li et al., 2020). In contrast, in the current study, *Clostridium* fermentation was not detected in MF silage neither with nor without inoculants. The lowest *Lactiplantibacillus* and the highest *Weissella* levels were observed in MF silages without inoculants. This was consistent with the results of Wang et al. (2020), where *Lactobacillus*, *Weissella*, and *Pediococcus* were dominant bacterial genera in alfalfa, and their mixed silages with corn. This process is quite variable, reflecting differences in environmental factors, such as crop species, climate, geographical location, and type of fertilizer applied (Gibson, 1965). Generally, both *Clostridium*-dominated fermentation and *Weissella-*dominated fermentation resulted in lower lactic acid content and higher pH in alfalfa silage and higher-ratio alfalfa silages with Napier grass.

In April 2020, the published genome data of *Lactobacillus* was analyzed, thus completing the research on important taxonomic changes of *Lactobacillus*. In the resulting new taxonomy, the genus *Lactobacillus* was re-divided into 25 genera (including 23 new genera and a revision of *Paralactobacillus*), and bacterial species with changing taxonomic statuses were described (Zheng et al., 2020). In the current study, *Lactobacillus* was mainly divided into *Lactiplantibacillus*, *Lacticaseibacillus*, *Companilactobacillus*, and *Levilactobacillus*. The type species of *Lactiplantibacillus* is *Lactiplantibacillus plantarum* comb. nov. *Lactiplantibacillus* was previously referred to as the *L. plantarum* group, with the type species *Lacticaseibacillus casei* comb. nov., and *Lacticaseibacillus* was previously referred to as the *L. casei* group (Zheng et al., 2020). For *Companilactobacillus*, the type species is *Companilactobacillus alimentarius* comb. nov. and *Companilactobacillus* was previously referred to as the *L. alimentarius* group. The type species of the *Levilactobacillus* genus is *Levilactobacillus brevis* comb. nov., and *Levilactobacillus* was previously referred to as the *L. brevis* group (Zheng et al., 2020). Through these classifications, a more detailed classification of *Lactobacillus* can be achieved, which can deepen the understanding of their role in silage fermentation, compared with the collective referral of LAB with different metabolic pathways and fermentation efficiencies as *Lactobacillus*. In the current study, *Lactiplantibacillus* was the most dominant bacterial genus in all silages, the abundance of which directly reflects the lactic acid content and pH in the silage. The abundance of *Lactiplantibacillus* increased significantly in silages treated with IN compared with CK (Figures 1A,B). The top three genera were *Lactiplantibacillus*, *Lacticaseibacillus*, and *Levilactobacillus*. This shows that *Lactobacillus* spp. in silages treated with IN has an absolute advantage. As a homofermentative LAB, *Lactiplantibacillus* has a high acid production efficiency, can effectively reduce the pH of silage, and even plays a positive role in alfalfa silage and high proportion alfalfa mixed silage. This result is consistent with Yang et al. (2018). Interestingly, inoculants responded differently to mixed silage at different mixing proportions (Figure 2). In Napier grass silage and mixed silage with a high proportion of Napier grass (M0 and M3), IN and CO inoculation treatments showed absolute dominance of *Lactobacillus* spp., including *Lactiplantibacillus*, *Companilactobacillus*, and *Levilactobacillus*, especially the silage inoculated with IN, where *Lactobacillus* spp. had an abundance of 75%. With increasing proportion of alfalfa in mixed silage, the abundance of *Lactobacillus* spp. decreased gradually, and the abundance of *Weissella* increased (Figures 2C–E). Surprisingly, the abundances of *Lactiplantibacillus* and *Lacticaseibacillus* in silages inoculated with IN remained at a relatively high level compared with CK and CO-treated silage. In addition to the fact that a higher proportion of alfalfa may result in higher buffer energy, making it difficult for silage to ferment, another reason may be related to the different DM contents resulting from different mixing proportions. Benjamim da Silva et al. (2022) found that whole-plant corn, harvested at low DM, showed a more modest proliferation of culturable LAB, which led to greater concentrations of lactic and acetic acids in silage. Kung et al. (2018) also reported that the concentrations of acetic and lactic acids are negatively correlated with DM content. These results showed that the DM content of mixed silage increased with increasing alfalfa proportion, making it more difficult for silage to produce lactic acid and acetic acid. Also, among IN and CO silages, the abundance of *Lactobacillus spp.* in M5, M7, and MF became inconsistent. These findings suggest that it was more difficult for the inoculant to compete if the DM content in the forage was high. A possible reason for the relatively inferior development of the inoculant in higher DM silage is the greater competitive pressure, especially because of competition with *Weissella*.

Although M0, M3, and M5 silages treated with IN can be pooled, these three groups cannot be easily separated from each other (Figure 3B). The possible explanation is that the effect of inoculants on silage treated with IN was more consistent at higher Napier grass ratios, but the effect of inoculants differs with increasing alfalfa ratio. LEfSe analysis showed that more biomarkers were *Lactobacillus spp.* in IN and CO treated silages. Especially in M5, M7, and MF silages, *Lactiplantibacillus*, *Levilactobacillus*, and *Lacticaseibacillus* were biomarkers in silages treated with IN. These results also showed that IN inoculation played a positive role in mixed silage with higher alfalfa proportion.

## Conclusion

A higher alfalfa mixing ratio improved the nutrient value of mixed silage with Napier grass, but also increased fermentation difficulty. With increasing alfalfa proportion, the abundance of *Weissella* increased rapidly. Inoculation with selected LAB improved the fermentation quality of mixed silage by increasing the abundance of *Lactiplantibacillus*. In conclusion, considering the balance of nutrients and fermentation, mixing Napier grass with alfalfa at ratios of 7:3 and 5:5 is optimal. If a higher proportion of alfalfa must be used, the use of LAB-inoculants is recommended to ensure sufficient fermentation.

## Data availability statement

The datasets presented in this study can be found in online repositories. The names of the repository/repositories and accession number(s) can be found at: https://www.ncbi.nlm.nih.gov/, PRJNA884491.

## Author contributions

HG and HL conceptualization. HG methodology, software, validation, formal analysis, writing – original draft preparation, visualization, and project administration. HG, HL, LG, and SC investigation. SC resources. HG and YH data curation. HG, ZJ, WL, and XW writing – review and editing. QZ supervision. HG and QZ funding acquisition. All authors contributed to the article and approved the submitted version.

## Funding

This work was supported by Qinghai Provincial key research and development program (grant no. 2022-NK-130), the Southwest Minzu University Research Startup Funds (grant no. RQD2022032), the Fundamental Research Funds for the Central Universities (ZYN2022054), China Forage and Grass Research System (CARS-34), and Key Laboratory of Superior Forage Germplasm in the Qinghai-Tibetan Plateau (2020-ZJ-Y03).

## Conflict of interest

The authors declare that the research was conducted in the absence of any commercial or financial relationships that could be construed as a potential conflict of interest.

## Publisher’s note

All claims expressed in this article are solely those of the authors and do not necessarily represent those of their affiliated organizations, or those of the publisher, the editors and the reviewers. Any product that may be evaluated in this article, or claim that may be made by its manufacturer, is not guaranteed or endorsed by the publisher.

## Footnotes

^1^ http://www.arb-silva.de/

## Abbreviations

DM: dry matter
CP: crude protein
WSC: water-soluble carbohydrates
NDF: neutral detergent fiber
ADF: acid detergent fiber
LAB: lactic acid bacteria
